# *RasGrf1* deficiency delays aging in mice

**DOI:** 10.18632/aging.100279

**Published:** 2011-03-14

**Authors:** Consuelo Borrás, Daniel Monleón, Raul López-Grueso, Juan Gambini, Leonardo Orlando, Federico V. Pallardó, Eugenio Santos, José Viña, Jaime Font de Mora

**Affiliations:** ^1^ Department of Physiology, School of Medicine, University of Valencia, E46010, Valencia, Spain; ^2^ Fundación de Investigación del Hospital Clínico Universitario de Valencia / INCLIVA, E46010, Valencia; ^3^ Centro de Investigación Príncipe Felipe, 46012 Valencia, Spain; ^4^ Centro de Investigación del Cáncer (USAL-CSIC), University of Salamanca. Campus Miguel de Unamuno s/n. 37007, Salamanca, Spain

**Keywords:** Longevity, Ras, metabolism, GEF, IGF-1, positron emission tomography

## Abstract

RasGRF1 is a Ras-guanine nucleotide exchange factor implicated in a variety of physiological processes including learning and memory and glucose homeostasis. To determine the role of RASGRF1 in aging, lifespan and metabolic parameters were analyzed in aged *RasGrf1^−/−^* mice. We observed that mice deficient for *RasGrf1^−/−^* display an increase in average and most importantly, in maximal lifespan (20% higher than controls). This was not due to the role of Ras in cancer because tumor-free survival was also enhanced in these animals. Aged *RasGrf1^−/−^* displayed better motor coordination than control mice. Protection against oxidative stress was similarly preserved in old *RasGrf1^−/−^*. IGF-I levels were lower in *RasGrf1^−/−^* than in controls. Furthermore, SIRT1 expression was increased in *RasGrf1^−/−^* animals. Consistent with this, the blood metabolomic profiles of *RasGrf1*-deficient mice resembled those observed in calorie-restricted animals. In addition, cardiac glucose consumption as determined PET was not altered by aging in the mutant model, indicating that *RasGrf1*-deficienct mice display delayed aging. Our observations link Ras signaling to lifespan and suggest that *RasGrf1* is an evolutionary conserved gene which could be targeted for the development of therapies to delay age-related processes.

## INTRODUCTION

RASGRF1 is a guanine nucleotide-releasing factor for RAS that is activated by calmodulin-mediated Ca^2+^ influx [1] as well as G-protein coupled receptors [2, 3]. RASGRF1 serves as an *in vivo* activator for H-RAS and members of the R-RAS and RAC subfamilies [4]. Guanine nucleotide-releasing factors interact with the inactive RAS bound to GDP and catalyze the exchange of GDP for GTP, thereby activating RAS. In contrast with other RAS-GTP exchange factors, RASGRF1 is expressed mainly in pancreatic β-cells where it regulates β-cell mass and in specific brain regions including the hippocampus and hypothalamus, thus linking RASGRF1-dependent RAS activation to glucose homeostasis and neuronal function [5, 6].

Previous reports have demonstrated that *RasGrf1*-defcient mice display defects in learning and memory, although the explanation for these impairments remains controversial [5, 7]. Similar to *RasGrf1* null mice generated in a different strain [8], our knockout mice display reduced body size. Additionally, we observed that *RasGrf1*-deficiency causes hypo-insulinemia due to a reduction in β-cell proliferation and β-cell mass [6]. However, perhaps owing to the fact that the size of these animals is reduced, insulin levels are sufficient to compensate under normal conditions and thus, diabetes does not develop in this model. Therefore, RASGRF1-mediated signaling is important in the regulation of β-cell proliferation and glucose homeostasis.

RAS has been shown conclusively to exert a role in aging in yeast. Mutations that decrease the activity of the RAS/Cyr1/PKA pathway extend longevity and increase stress resistance in yeast by activating transcription factors Msn2/Msn4 and the mitochondrial superoxide dismutase [9]. It has also been reported that *Ras* genes are a major homeostatic device in the regulation of the lifespan of *S. cerevisiae*[10]. The longevity-modulating function of IGF-I in *C. elegans* displays a signal bifurcation involving the *Ras* ortholog Let-60, consistent with a role for RAS signaling downstream of IGF-IR. Attenuated insulin/IGF-I signaling (IIS) is hypothesized to mediate some of the anti-aging effects of calorie restriction in mice [11]. Importantly, mice heterozygous for the IGF-IR live longer and are resistant to oxidative stress [12]. Moreover, calorie restriction has been suggested to down-regulate pathways including m-TOR, AKT and RAS [13].

Several studies with cultures of mammalian cells have suggested that RAS might be involved in aging and age-related processes such as apoptosis. Apoptosis in neurons, T cells, and human epithelial cells is mediated by the activation of RAS [14][15, 16]. On the contrary, inhibition of RAS rescues PC12 cells from apoptosis [17]. Moreover, inhibition of RAS in these cells increases resistance to oxidative stress [18, 19].

However, few studies have addressed the potential role of RAS signaling in regulating lifespan in mammals [20]. Based on the previous data from our laboratory [6] as well as others [8], we hypothesized that *RasGrf1*-deficient mice could have increased longevity. We measured plasma IGF-I levels as it related to RAS and aging. We studied the expression of cytochrome c oxidase to determine whether oxidative stress is altered in *RasGrf1*-deficient mice. Finally, to test the idea that *RasGrf1* deficiency promotes beneficial metabolic changes, we compared the metabolomic profile of our *RasGrf1-* deficient mice with calorie restricted controls.

Our studies reveal that loss of *RasGrf1* (*RasGrf1-*KO) expands not only average but also maximal lifespan in mice. These mice display lower plasma levels of IGF-1, lower oxidative stress and a metabolic profile which resembles that observed in calorie-restricted wild-type animals. Thus, *RasGrf1* deficiency in mice promotes longevity consistent with the lifespan extension associated with loss of IGF-I signalling molecules from yeast to mammals.

## RESULTS

### Increased Average and Maximal Lifespan in *RasGrf1^−/−^* Mice

**Figure 1.**
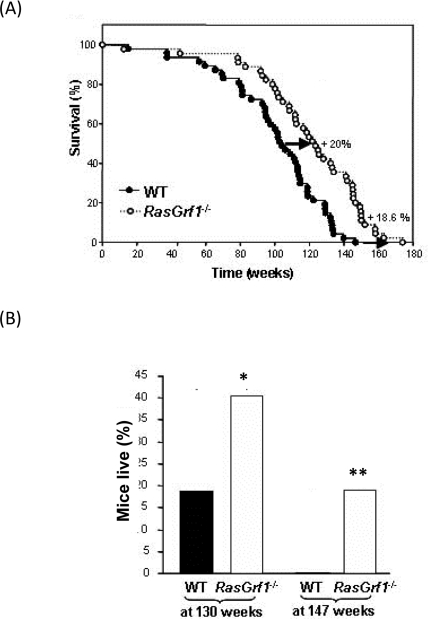
Delayed Ageing in *RasGrf1^−/−.^* (**A**) Survival curve of *RasGrf1^−/−^* male mice and of WT of the same genetic background. WT n = 47; *RasGrf1^−/−^* n = 45. Report the Kaplan - Meyer representation of the two groups (p<0.05). (**B**) Percentage of mice alive after a hundred and thirty weeks (*p<0.02) and a hundred and forty-seven weeks (**p<0.005) of the two cohorts. Maximal lifespan in wild type was a hundred and forty-seven weeks. Thus 20% of all the *RasGrf1^−/−^* cohort survived longer than the maximal lifespan of the wild types.

Survival curves revealed a marked increase (20%) in the average lifespan of *RasGrf1^−/−^* male mice (mean values WT: 100.5±4.2 weeks and *RasGrf1^−/−^*: 120.7±4.7 weeks; median WT: 104 weeks; *RasGrf1^−/−^*: 124 weeks) (p= 6×10^−5^, Logrank test). The effects of *RasGrf1* deficiency were also evident on maximal lifespan; WT mice lived up to 146.7 weeks whereas *RasGrf1* mutants lived as long as 174 weeks (Figure 1A). This increase of 18.6% to maximal lifespan supports a critical role for *RasGrf1* in longevity. In fact, 19,2% of *RasGrf1^−/−^* mice lived longer than the oldest WT (Figure 1B). The lifespan of our WT mice is within the range of variation reported for C57B1, 129 and hybrids [21-27].

One plausible explanation for these longevity promoting effects could be the role of RASGRF1 in RAS activation and hence, in cancer development. To test this possibility, we identified those animals that developed tumors and generated a tumor-free survival curve. Consistent with the curve generated for the total population, *RasGrf1^−/−^* without tumors displayed a 20% increase in average lifespan as compared with tumor-free control mice (mean values WT: 99±4.5 weeks and *RasGrf1^−/−^*: 119±4.9; median WT: 102 weeks; *RasGrf1^−/−^*: 121 weeks) (p= 0.0003, Logrank test) (Figure 1B). Thus, the role of RAS in cancer is not the reason for the longer longevity of *RasGrf1^−/−^* mice. No obvious pathological differences were observed at death between WT and *RasGrf1^−/−^* mice. The substantial increase in average and maximal lifespan suggests *RasGrf1* as a novel age-associated gene.

### *RasGrf1^−/−^* Mice Show Better Motor Coordination than Controls

**Figure 2.**
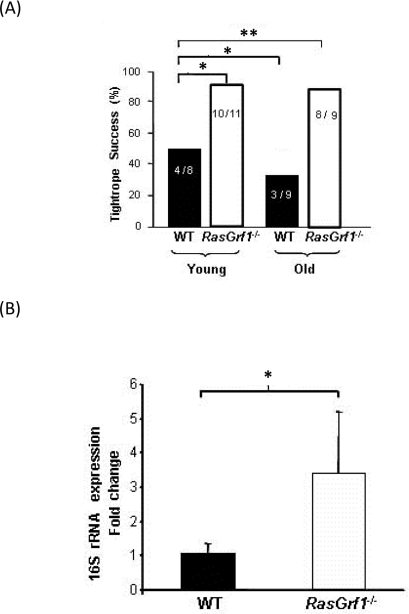
Increased Neuromuscular Coordination Coincided With Biomarkers of Aging in *RasGrf1^−/−^* Mice (**A**) Neuromuscular coordination was quantified as the percentage of male mice that successfully passed the tightrope test. Numbers within the bars indicate animals that passed the test divided by the total number of animals which were subjected to the test. Young animals were 4-6 months old, and old animals were 20-22 months old. Significance is shown as *p<0.05; **p<0.01 vs. WT. (**B**) Liver extracts from male mice were used to assess 16S rRNA expression in 4-6 months old animals. Expression of 16S rRNA was significantly higher in *RasGrf1^−/−^* mice. WT n = 4; *RasGrf1^−/−^* = 3; *p=0.03.

Maintaining good motor coordination in old age is important to ensure that living longer is accompanied by an adequate quality of life. Young *RasGrf1^−/−^* mice displayed superior motor coordination in the tightrope test as compared to WT controls (Figure 2A). Whereas motor coordination of WT mice declined significantly with age, old *RasGrf1^−/−^* maintained a level of motor coordination that was similar to young animals (Figure 2A). Thus, our analysis suggests that *RasGrf1^−/−^* mice not only live longer but they are less prone to frailty.

### Expression of 16S rRNA is higher in *RasGrf1^−/−^* mice than in control mice

We examined the expression levels of 16S rRNA, which decreases significantly with age [28, 29] and oxidative stress [30]. 16S RNA levels were much higher in liver of *RasGrf1^−/−^* than WT of the same age (Figure 2B). This result is consistent with the increased lifespan observed in *RasGrf1^−/−^* mice.

### *RasGrf1^−/−^* Mice Display Less Oxidative Stress

**Figure 3.**
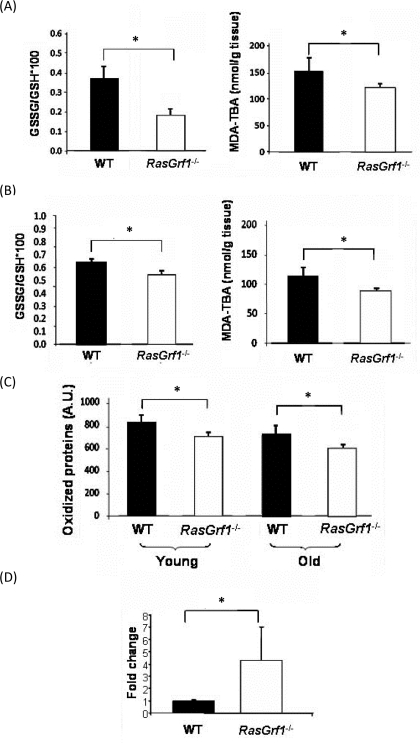
Decreased Oxidative Stress in *RasGrf1^−/−^* Mice (**A**) Oxidative stress was measured in brain from 4-6 months old animals. Glutathione redox ratio was significantly higher in WT (n=7) than in *RasGrf1^−/−^* male mice (n=8). Malondialdehyde (MDA) levels were significantly lower in brain of *RasGrf1^−/−^* mice (n=8) than in that of WT (n=7). MDA was measured by the formation of the aduct malondialdehyde-thiobarbituric acid (MDA-TBA) (*p<0.05). (**B**) Oxidised glutathione and malondialdehyde levels were significantly lower in liver of *RasGrf1^−/−^* male mice. n for WT = 7 and for *RasGrf1^−/−^* = 8 (*p<0.05) (4-6 months old). (**C**) Oxidised proteins in young (4-6 months old) and old (20-22 months old) WT mice were significantly higher than in *RasGrf1^−/−^* male mice (*p<0.05). These were measured by western blot with antibodies directed against aldehydes in proteins. (**D**) Cytochrome c oxidase expression is significantly higher in *RasGrf1^−/−^* than in WT male mice of 4-6 months old (*p=0.037). Total RNA from selected tissues was isolated and used to quantify cytochrome c oxidase by real time RT-PCR.

To analyze the contribution of the free radicals to the increased lifespan associated with *RasGrf1* deficiency, we assessed three of the critical parameters of oxidative stress: glutathione, protein oxidation, and malondialdehyde (MDA) which is a measurement of lipid peroxidation. Oxidative stress diminishes the levels of reduced-glutathione (GSH), the most abundant non- protein antioxidant in cells, to form the disulphide bond-dimer (GSSG). The GSSG/GSH ratio was significantly lower in brain and liver of *RasGrf1^−/−^* mice as compared with controls (Figure 3A,B upper panels), indicating that *RasGrf1^−/−^* mice are subjected to less oxidative stress. Additionally, lipid peroxide levels were significantly lower in *RasGrf1^−/−^* mice (Figure 3A,B lower panels). Since aging is characterized by the accumulation of oxidized proteins due to increased protein damage and/or decreased elimination, we evaluated this parameter in liver of young and old animals. The overall oxidized protein levels in liver of *RasGrf1^−/−^* were significantly lower than in age-matched WT mice (Figure 3C). These results support the idea that loss of *RasGrf1* protects against oxidative damage and hence, contributes to a younger phenotype. We next examined a possible molecular basis of these findings. Low levels of cytochrome c oxidase, the terminal enzyme in the mitochondrial electron transport chain, increase the production of reactive oxygen species (ROS) [31]. Interestingly, the expression levels of cytochrome c oxidase were significantly higher in liver of *RasGrf1^−/−^* mice (Figure 3D). Thus, disruption of the RAS signaling via deletion of *RasGrf1* up-regulates the expression of cytochrome c oxidase, providing at least one explanation for the lower oxidative stress in *RasGrf1^−/−^* mice.

### Higher Glycogen Hepatic Content In *RasGrf1^−/−^* Mice

**Figure 4.**
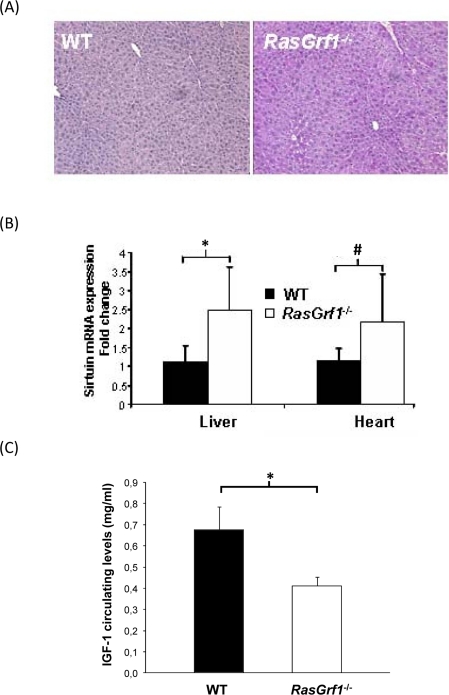
Metabolic Analysis of *RasGrf1^−/−^* Mice (**A**) Liver of *RasGrf1^−/−^* male animals (4-6 months old) contains a significant amount of glycogen after 24 hours of fasting contrasting with the complete absence in WT animals (4-6 months old). PAS staining was quantified using color deconvolution with ImageJ software (Broken Symmetry Software). Values represent relative staining of three different sections (*p=0.01 determined by a paired T-test). (**B**) Sirtuin mRNA expression in liver and heart of *RasGrf1^−/−^* male mice is significantly higher than in WT mice (*p=0.033; #p=0.035). Wild-type n = 4, *RasGrf1^−/−^* n = 3 (4-6 months old). (**C**) IGF-I plasma levels was measured in 3-5 months old male mice by RIA revealing a 30% reduction in *RasGrf1^−/−^*. Wild-type n = 10, *RasGrf1^−/−^* n = 9, *p<0.001.

Aging is characterized by a diminished response to stress [32]. Maintenance of glycogen storage is important in the stress response [33]. We found that hepatic glycogen in WT mice was virtually absent after 24 hours of fasting whereas *RasGrf1^−/−^* mice maintained significant glycogen levels (Figure 4A). Our findings reveal a glycogen-sparing effect mediated by loss of a specific gene. Maintenance of relatively high levels of glycogen in fasting would be expected to convey a metabolic advantage in response to stress during aging.

### Increased Expression of SIRT1 in *RasGrf1^−/−^* mice

Sirtuins have appear to play a role in determining longevity [34], but their importance in mammalian lifespan is not clear (Herranz & Serrano 2010). We observed that the levels of SIRT1 are increased in the liver and heart of *RasGrf1^−/−^* mice (Figure 4B). High sirtuin might explain the glycogen sparing effect that we have shown above (see discussion).

### IGF-1 Levels Are Lower in *RasGrf1^−/−^* Mice

Long-lived animals display low IGF-1 levels [35-37]. We observed a significant decrease of IGF-1 in *RasGrf1^−/−^* mice when compared to controls (Figure 4C).

Therefore, this could explain, at least in part, the increased longevity of *RasGrf1^−/−^* mice. Consistent with the low IGF-1 levels, somatic growth was also reduced in *RasGrf1^−/−^* mice (See Figure S3).

**Figure 5.**
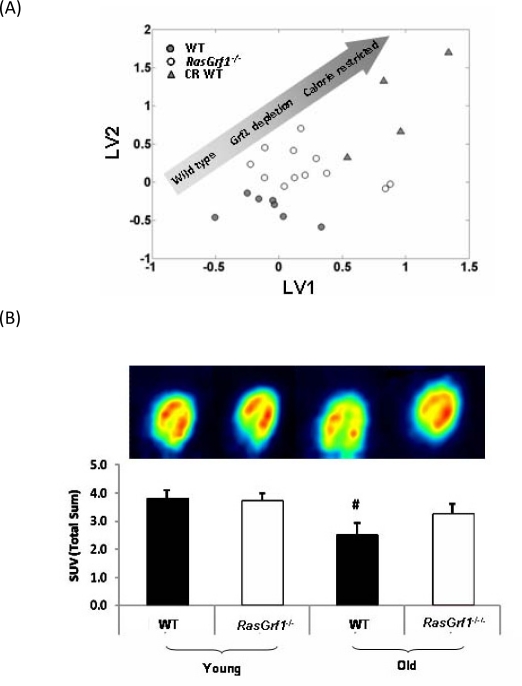
Analysis Of Metabolic Parameters in *RasGrf1* Deficient and Control Mice (**A**) Metabolomic analysis reveals a metabolic shift of *RasGrf1^−/−^* fed male mice towards caloric restricted WT (4-6 months old). Multivariate analysis (PLS - DA) of NMR spectra of blood plasma showing global metabolic profiles of WT, *RasGrf1^−/−^*, and calorie restricted WT mice. The graph represents the scores plot of the PLS-DA model for discrimination between WT and *RasGrf1^−/−^*. The graph also shows the metabolic profile of WT animals on caloric restriction projected over the PLS-DA latent space. Each symbol represents an animal. (**B**) Glucose up-take *in vivo* by heart of WT and *RasGrf1^−/−^* male mice. Positron emission tomography (PET) analysis was used to estimate *in vivo* glucose uptake in young (4-6 months old) and old (20-22 months old) animals. The image is the result of a representative experiment. Histograms represent the means of glucose up-take measured in four animals in each group. Wild type n = 7, *RasGrf1^−/−^* n = 8; #p<0.01.

### Metabolomics Reveal that the Metabolic Profile of *RasGrf1^−/−^* Mice Resembles Calorie Restricted Mice

To gain further insight into the mechanisms that promote increased average and maximal lifespan *RasGrf1^−/−^* mice, we performed metabolomic studies in which we compared *RasGrf1^−/−^* animals with WT animals, and calorie-restricted WT animals. Figure 5A presents the global metabolic profile of *RasGrf1^−/−^* and WT animals as measured by NMR. The distribution of the three groups represented in the plot indicates that the metabolic profile exhibited by *RasGrf1^−/−^* animals is shifted towards that of the CR animals. The multivariate analysis reveals a set of metabolites with significant variations between WT and *RasGrf1^−/−^* mice (Figure S1). Most of these metabolic differences also occur in CR such as an increase in the levels of polyunsaturated fatty acids (PUFA; 14%, p=0.002), and decrease in glucose levels (10%, p=0.03) in *RasGrf1^−/−^*.

**Figure 6.**
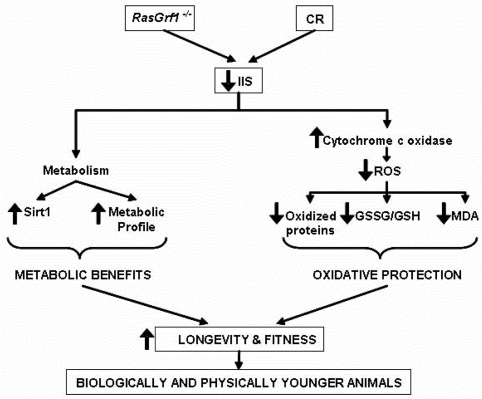
Schematic Representation of the Molecular and Physiological Characteristics of *RasGrf1^−/−^* Animals *RasGrf1−/−* results in an increased longevity which is mediated by a lower insulin /IGF signaling (IIS) which eventually leads to metabolic benefits and in lower ROS production and subsequent oxidative protection. These two independent changes converge in a notable increase in longevity and fitness.

### Glucose Uptake *In Vivo* Is Maintained in Aged *RasGrf1^−/−^* Mice But Not in Control Mice

We used positron emission tomography (PET) to analyze *in vivo* glucose uptake in young and old animals since reduced glucose uptake is associated with aging [38]. Glucose uptake by heart of control animals was significantly lowered with age. Surprisingly, this loss of glucose consumption did not occur with aging in *RasGrf1^−/−^* animals (Figure 5B). Thus, although the critical parameter of glucose incorporation in cardiac tissue declined considerably in aged WT controls, it was preserved in *RasGrf1^−/−^* animals.

## DISCUSSION

Interest in genetic components that modulate longevity in mammals has grown in recent years [39, 40]. According to the National Institute on Aging (NIA), the genetically heterogeneous mouse model was deemed to be the most suitable for longevity studies [41], even if this increases the genetic variability of each mouse and decreases the power of attaining statistical significance [39]. We have recently reported that over-expressing two (p53/p16) [42] or three (p53/p16/telomerase) [43] genes results in significant increases in average lifespan but without increasing maximal lifespan. Here we report a significant increase in maximal lifespan by the deletion of *RasGrf1* in inbred mice with a heterogeneous background. Increasing maximal lifespan is important because it indicates a fundamental change in the aging process. Improvements in lifestyle conditions usually result in increases to average but not maximal lifespan [32]. Extrapolating to humans, curing all diseases would result in an increase of less than 20% in average lifespan without changing maximal lifespan [32].

Motor coordination is an important determinant of frailty, a major geriatric syndrome [44, 45]. Ladiges et al. have emphasized the importance of finding genetic modifications that improve patho-physiological functions associated with aging, such as motor coordination [39]. We have found that motor coordination is improved in the *RasGrf1* deficient mice (Figure 2A), thus indicating that these animals not only live longer but are less prone to frailty.

Based on genomic scanning with methylation sensitive enzymes, *RasGrf1* was postulated as a paternally imprinted gene in the mouse [46]. This was confirmed by the subsequent generation of *RasGrf1-*deficient mice; studies of heterozygous animals demonstrated that *RasGrf1* is an imprinted gene regulated by the male and modulates postnatal growth because it functions after, rather than before, birth [8]. The imprinted nature of *RasGrf1* was further validated by the characterization of two nonsense mutations that when paternally transmitted caused reduced body weight during postnatal development [47]. A more recent study with bi-maternal mice revealed that the sperm genome has a detrimental effect on longevity, most likely due to the repression of *RasGrf1* in this model [48]. In contrast to other imprinted genes that are implicated in fetal growth, *RasGrf1* regulates postnatal growth. RASGRF1 is expressed after birth in the hypothalamus of wild-type mice, but not in the pituitary, where it indirectly regulates the synthesis of growth hormone [8]. Many genes that modulate longevity directly or indirectly target mitochondrial function. Mitochondria are inherited via the female parent. Similar to other paternally expressed genes, *RasGrf1* is involved in growth stimulation, whereas maternally expressed genes are responsible for growth suppression [8, 49]. Hence, our results describe a longevity-related gene which is solely determined by the male parent.

Circulating IGF-I levels and body size are important determinants of lifespan [37]. A previous publication reported reduced IGF-I circulating levels and adult body size in mice deficient for *RasGrf1^−/−^*[8]. However, this model was generated in pure 129 sv genetic background which is distinct from our own *RasGrf1^−/−^* mice which are genetically heterogeneous mice. Since genetic background can exert a profound effect in parameters such as metabolism and growth ratio, we measured IGF-I circulating levels in our own *RasGrf1^−/−^* model. In agreement with the other knockout model, circulating IGF-I was reduced by 39% in our *RasGrf1^−/−^* mice (Figure 4C). Hence, *RasGrf1^−/−^* mice display both low insulin and IGF-I levels that may significantly impact systemic IIS. In agreement with low IGF-1 levels, *RasGrf1^−/−^* mice display reduced body weight (See Figure S3). We previously reported that food intake, circulating leptin levels and leptin receptor expression in the hypothalamus are very similar between WT and *RasGrf1^−/−^* mice [6], suggesting that food intake is not the mechanism for the longer lifespan of *RasGrf1^−/−^* mice. Therefore, the increase in lifespan observed in *RasGrf1^−/−^* mice is most probably due to their lower insulin and IGF-1 levels.

Lower serum IGF-I and insulin levels are characteristic of animals subjected to caloric restriction and are associated with increased SIRT1 expression and reduced oxidative stress [50, 51]. In this paper we also show higher levels of SIRT1 expression (Figure 4B) and reduced oxidative stress (Figure 3) in *RasGrf1^−/−^* mice. Metabolomic results also indicate that *RasGrf1^−/−^* mice could resemble in some way calorie restricted mice, as it is shown in Figure 5. The positive effects of CR on lifespan extension may involve the down-regulation of pathways, including mTOR, AKT, and RAS [13]. Our results demonstrate a novel physiological function of the RAS modulator RASGRF1 in extending both median and maximum lifespan.

SIRT1 deacetylates PPARγ and its co-activator PGC1α, thereby promoting fat mobilization and increasing mitochondrial size and number [52-55]. Activation of SIRT1 stimulates hepatic energy expenditure by increasing the degradation of fatty acids [56]. Conversely, under fasting conditions (e.g. CR), glycolytic genes are repressed through a mechanism that involves PGC1a and SIRT1 [55]. Therefore, increased SIRT1 expression would be expected to enhance this repression and promote the accumulation of glucose reservoirs in the form of glycogen. Indeed, increased hepatic SIRT1 expression correlated with enhanced glycogen accumulation in the liver of fasted *RasGrf1^−/−^* mice (Figure 4A). Although glycogen storage is regulated through a plethora of conditions, the fact that *RasGrf1^−/−^* mice exhibit higher liver glycogen levels could also be consistent with increased SIRT1 levels mediated by FoxO1-dependent transcriptional activity [57]. In contrast, hepatic glycogen levels after a 24 hour fast were undetectable WT control mice.

Glucose uptake favors a metabolic phenotype that protects against age-associated susceptibility to ischemic injury [58]. Therefore, preservation of cardiac glucose uptake may represent yet another physiological advantage for the *RasGrf1^−/−^* extended lifespan. Decline in cardiac performance with age is prevented in *Drosophila* by disruption of insulin receptor signaling specifically in the heart [59]. Additionally, higher plasma concentrations of PUFA detected in *RasGrf1^−/−^* are also expected to have beneficial effects for cardiovascular disease and lifespan.

The increased longevity of *RasGrf1^−/−^* mice may be explained by the low IGF-1 levels [37]. The physiological explanation linking these low levels and *RasGrf1* deficiency at the cellular level is very difficult to obtain because *RasGrf1* is only expressed in hippo-campus, hypothalamic, β-pancreatic and mature adipocytes. Adipocytes do not synthesize IGF-I. Hypothalamic and β-pancreatic cell do but they cannot be obtained in sufficient amounts to perform cell- signaling studies. Cells usually used in these kinds of studies, like mouse embryonic fibroblasts do not express *RasGrf1.* The involvement of *Ras*, but not *RasGrf1*, in longevity has been extensively studied by Longo and coworkers [20, 60, 61]. These authors have shown in yeast, that RAS regulates stress resistance and longevity by activating transcription factors Msn2/Msn4 and the mitochondrial antioxidant enzyme superoxide dismutase (Sod2) [60]. We also observed less oxidative stress in the *RasGrf1* deficient mice, and we show that the mitochondrial antioxidant enzyme which is up-regulated is cytochrome c oxidase (Figure 3). Thus, the increased longevity and resistance to stress in *RasGrf1* deficient mice show that the negative influence of the RAS pathway may be conserved mechanism from yeast to mammals.

Moreover, based in a genome-wide association study, Sebastiani and co-workers found a set of 150 single nucleotide polymorphisms (SNPs) that can predict exceptional longevity (EL) in humans [62]. This novel approach for predicting EL provided valuable information regarding genes that contribute to EL. *RasGrf1* was found to be one of such genes. We have demonstrated that deficiency in *RasGrf1* expands not only average but also maximal lifespan, thus revealing to be a longevity-conserved gene in rodents and humans. Recently, Kawahara and Kono found that bi-maternal mice (i.e. “mice without a father”) exhibited increased longevity and this was attributed it to lack of *RasGrf1* [48].

In summary, our results demonstrate that *RasGrf1^−/−^* display less oxidative stress than control mice and also have lower insulin/IGF-1 circulating and higher sirtuin expression. Thus, the increase in maximal longevity in this model may be explained by the fact that two main theories of aging (the free radical and the IGF-I theories of aging) are at work in the *RasGrf1^−/−^* mice (Figure 6).

## MATERIALS AND METHODS

### Mice

Male wild-type (WT) and *RasGrf1^−/−^* mice were maintained in ventilated cages and survival function was estimated from lifespan data.

Handling, supervision and experimentation with mice was done in accordance to the Guidelines for Humane Endpoints for Animals Used in Biomedical Research. Mice were housed at the pathogen-free barrier area of the Prince Felipe Research Centre. Moribund criteria was established and approved by the Ethical Committee at the Prince Felipe Research Centre. These criteria were based on the suggested clinical endpoints for aging mice according to McGill University's Ethics Subcommittee (www.mcgill.ca/files/researchoffice/Aging-mice-6.doc). Briefly, animals were assigned a health score based on their general appearance, wounds, prolapses, size and location of tumors, etc., as described in Table S1. Depending of the score, animals were monitored daily when symptoms were minor. In more critical cases (loss of vital functions or a score over 18 points) animals were immediately euthanized.

*RasGrf1^−/−^* mice were originally generated in 1998 at NCI facilities in Frederick (MD). Transfections were done in RW-4 cells (derived from 129SvJ mouse strain) to obtain the gene deletion. Cells were injected into C57BL/6N blastocysts to generate chimera animals that were further backcrossed twice with C57Bl/6N mice to generate both, wild-type and *RasGrf1−/−* mice. No further backcrosses were done with C57Bl/6N.

From 1998 to present, the colony has been routinely propagated by homozygous matings [6]. However, animals for the longevity study were generated by crossing heterozygous animals in order to obtain sibling controls.

Diet provided was Harlan Teklad Global Maintenance 2014 (Harlan Italy). Animals used for the longevity curve were solely used for this study. Animals were allowed to die naturally except when their vital constants were compromised and were further euthanized.

### Food intake

To measure food intake in mice, 5 male RasGrf1−/− mice and 5 age-matched (2 months old) wild-type mice were weight and kept in separate cages. Mice were provided with powder food (2014 Global Rodent Maintenance Powder, Harlan Teklad) and intake of the diet was measured by successive daily weighings of the food container to an accuracy of 0.01 g on a top-loading electronic balance (Sartorius). Food intake was not significative different between these mice [6]; wild-type 131±6 and *RasGrf1^−/−^* 141±7 mg/g body weight/day; p=0.4).

### Tightrope test

The tightrope test is a widely used and extensively validated behavioural marker of ageing [63]. We performed the test with slight variations: mice were placed on a bar of circular section (60 cm long and 1.5 cm diameter) and the test was considered successful when a mouse did not fall during a period of 60 s in at least one out of five consecutive trials.

### Quantitative real-time RT-PCR

Total RNA from tissues was extracted with Trizol (Life Technologies). Messenger RNA levels were measured by quantitative real-time polymerase chain reaction using the Tth DNA polymerase kit (Roche Diagnostics-Boehringer Mannheim), as described by the manufacturer. Real-time quantitation of mRNAs for 16S rRNA and situins relative to GAPDH mRNA was performed using the iCycler (Bio-Rad, Hercules, CA) with SYBR Green I detection. Target cDNAs were amplified in separate tubes: 10 min at 95°C, then 40 cycles of denaturation (95°C for 30 s) and annealing and extension (at 62°C for 1 min per cycle). The increase in fluorescence was measured during the extension step. The threshold cycle (Ct) was determined, and the relative gene expression was expressed as fold change = 2^(-^^ΔΔ^^Ct)^. Specific primers used were 16S rRNA, 5′-GACGAGAAGACCCTATGGAG-3′ and 5′-AGAAACCGACCTGGATTGC-3′; SIRT-1 5′-CCAGATCCTCAAGCCATG TT-3′ and 5′-TTGCAGGAATCCAAAGGATC-3′ and GAPDH, 5′- CCTGGAGAAACC TGCCAA GTA TG-3′ and 5′-GGTCCTCAGTGTAGCCCAAGATG-3′. The error bars correspond to the relative error of the 2^(-^^ΔΔ^^Ct)^ values.

### Measurement of oxidative stress parameters and IGF-I levels

Glutathione ratio (GSSG/GSH*100) was determined by high-performance liquid chromatography (HPLC) as described [64]. Lipid peroxidation was determined as accumulation of MDA, which was detected by HPLC as an MDA–thiobarbituric acid adduct [65]. Oxidative modification of total proteins was assessed by immunoblot detection of protein carbonyl groups using the ‘OxyBlot’ protein oxidation kit (Intergen) following the manufacturer's instructions. The procedure to quantify total protein carbonyls with the OxyBlot kit was densitometry of the oxyblot and of the Ponceau staining, followed by finding the ratio between the total density in the oxyblot and the total density in the Ponceau. Serum levels of IGF-I were measured using retro-orbital blood and the Mouse/Rat IGF-I kit from Diagnostic Systems Laboratories.

### Determination of heart glucose consumption *in vivo.*

Mice were deprived of food for 8–14 h before 18F-2-fluor-2-deoxiglucose (18F-FDG) injection. 18F-FDG (5.8-11.1 MBq) was injected intraperitoneally after anesthesia with isoflurane (1.5–2% in100% oxygen, IsoFlo; Abbott Laboratories). PET was started 60 min after 18F-FDG injection as described in [66]. 18F-FDG was synthesized as previously described [67]. The administered dose (FDG activity) was indeed corrected for body weight. We acquired 20-min static images 60 min after injection of 18FFDG. The biodistribution of 18F-FDG by the heart was compared between all the studied groups. The PET images were obtained with the Albira small animal PET (ONCOVISION, GEM-Imaging). Regions of interest were manually drawn over the brain and heart with PMOD software. Tracer uptake by heart was quantified as SUV (Standardized Uptake Value, Total Sum).

### Storage, preparation and ^1^H NMR spectroscopic analysis of blood serum

Mice blood sera were stored at -80C and thawed before use. For NMR analysis, 20 μl of serum were mixed with 2 μl of D_2_O (as a field lock). A total of 20 μL of the mixture of each sample was then transferred into a 1 mm high quality NMR capillary individually. All ^1^H NMR spectra were acquired using a standard one-dimensional pulse sequence with water suppression (Bruker Avance 600 spectrometer operating at 600.13 MHz with a 1mm ^1^H/^13^C/^15^N TXI probe). A total of 256 FIDs (free induction decay) were collected into 64k data points with a spectral width of 14 ppm and the recycle delay (RD) of 1 s. Water signal was saturated with a weak irradiation during the recycle delay. Before Fourier transformation, the free induction decay was multiplied by a 0.3 Hz exponential line broadening. Spectral chemical shift referencing on the Alanine CH_3_ doublet signal at 1.475 ppm was performed in all spectra. Spectral regions between 0.5 and 4.5 ppm and between 5.5 and 9.5 ppm were binned in segments of 0.01 ppm width (6 Hz) for multivariate analysis. We normalized the binned data to total spectral area. We used available spectral databases and 2D NMR experiments to aid structural identification of relevant metabolites. All spectra were processed using MNova (MestreLab, Santiago de Compostela, Spain) and transferred to MATLAB® (MathWorks Inc, 2006) using in-house scripts for data analysis. Signals belonging to selected metabolites (Glucose, signal at 3.79 ppm and PUFA signal at 2.70 ppm) were integrated and quantified using semi-automated in-house MATLAB peak-fitting routines. These fitting routine were based on Levenburg-Marquard optimization procedures. The target function for the optimization included experimental spectra measured for standard solutions of selected metabolites with complex multiplet patterns and theoretically generated Lorentzian-shape signals for those metabolites with simpler spectral patterns. One-way-analysis of variance (ANOVA) was used for the determination of statistical significance between group means of the corresponding integrals.

### Multivariate analysis of NMR spectra

We used PLS_Toolbox 5.0 (Eigenvector Research, WA, USA) for MATLAB® to build the PLS-DA models. PLS-DA is a classification technique that encompasses the properties of Partial Least Squares regression with the discrimination power of discriminant analysis [68]. The main advantage of PLS-DA models is that the main sources of variability in the data are modeled by the so-called latent variables (LVs), and consequently, in their associated scores and loadings, allowing the visualization and understanding of different patterns and relations in the data. Binned spectral regions for blood serum from normally fed WT and *RasGrf1^−/−^* mice were mean-centered for building a PLS-DA model. The PLS-DA model discriminating between WT and *RasGrf1^−/−^* was cross-validated by the leave-on-out method providing a cross-validation RMS of 0.3816 and a cross validation classification error of 0.0625. Q residual and Hotelling T^2^ for 95% interval of confidence were 0.5047 and 14.28 respectively. Then, we project the spectra of blood serum from calorie restricted WT mice to this PLS-DA latent space for evaluating metabolic proximity between groups.

### Glycogen content determination

Livers from 24 hours fasted animals were embedded in paraffin and 5μm sections were stained for glycogen by standard periodic acid Schiff's (PAS) protocol.

## SUPPLEMENTARY MATERIAL



## References

[R1] Farnsworth CL, Freshney NW, Rosen LB, Ghosh A, Greenberg ME, Feig LA (1995). Calcium activation of Ras mediated by neuronal exchange factor Ras-GRF. Nature.

[R2] Mattingly RR, Macara IG (1996). Phosphorylation-dependent activation of the Ras-GRF/CDC25Mm exchange factor by muscarinic receptors and G-protein beta gamma subunits. Nature.

[R3] Shou C, Wurmser A, Suen KL, Barbacid M, Feig LA, Ling K (1995). Differential response of the Ras exchange factor, Ras-GRF to tyrosine kinase and G protein mediated signals. Oncogene.

[R4] Santos E, Fernandez-Medarde A Rasgrf1.

[R5] Brambilla R, Gnesutta N, Minichiello L, White G, Roylance AJ, Herron CE, Ramsey M, Wolfer DP, Cestari V, Rossi-Arnaud C, Grant SG, Chapman PF, Lipp HP, Sturani E, Klein R (1997). A role for the Ras signalling pathway in synaptic transmission and long-term memory. Nature.

[R6] Font de Mora J, Esteban LM, Burks DJ, Nunez A, Garces C, Garcia-Barrado MJ, Iglesias-Osma MC, Moratinos J, Ward JM, Santos E (2003). Ras-GRF1 signaling is required for normal beta-cell development and glucose homeostasis. Embo J.

[R7] Giese KP, Friedman E, Telliez JB, Fedorov NB, Wines M, Feig LA, Silva AJ (2001). Hippocampus-dependent learning and memory is impaired in mice lacking the Ras-guanine-nucleotide releasing factor 1 (Ras-GRF1). Neuropharmacology.

[R8] Itier JM, Tremp GL, Leonard JF, Multon MC, Ret G, Schweighoffer F, Tocque B, Bluet-Pajot MT, Cormier V, Dautry F (1998). Imprinted gene in postnatal growth role. Nature.

[R9] Fabrizio P, Liou LL, Moy VN, Diaspro A, Valentine JS, Gralla EB, Longo VD (2003). SOD2 functions downstream of Sch9 to extend longevity in yeast. Genetics.

[R10] De Benedictis G, Carrieri G, Garasto S, Rose G, Varcasia O, Bonafe M, Franceschi C, Jazwinski SM (2000). Does a retrograde response in human aging and longevity exist?. Exp Gerontol.

[R11] Fontana L, Partridge L, Longo VD (2010). Extending healthy life span--from yeast to humans. Science.

[R12] Harrison DE, Strong R, Sharp ZD, Nelson JF, Astle CM, Flurkey K, Nadon NL, Wilkinson JE, Frenkel K, Carter CS, Pahor M, Javors MA, Fernandez E, Miller RA (2009). Rapamycin fed late in life extends lifespan in genetically heterogeneous mice. Nature.

[R13] Wei M, Fabrizio P, Hu J, Ge H, Cheng C, Li L, Longo VD (2008). Life span extension by calorie restriction depends on Rim15 and transcription factors downstream of Ras/PKA, Tor, and Sch9. PLoS Genet.

[R14] Henkemeyer M, Rossi DJ, Holmyard DP, Puri MC, Mbamalu G, Harpal K, Shih TS, Jacks T, Pawson T (1995). Vascular system defects and neuronal apoptosis in mice lacking ras GTPase-activating protein. Nature.

[R15] Gomez J, Martinez AC, Fernandez B, Garcia A, Rebollo A (1996). Critical role of Ras in the proliferation and prevention of apoptosis mediated by IL-2. J Immunol.

[R16] Hall-Jackson CA, Jones T, Eccles NG, Dawson TP, Bond JA, Gescher A, Wynford-Thomas D (1998). Induction of cell death by stimulation of protein kinase C in human epithelial cells expressing a mutant ras oncogene: a potential therapeutic target. Br J Cancer.

[R17] Ferrari G, Greene LA (1994). Proliferative inhibition by dominant-negative Ras rescues naive and neuronally differentiated PC12 cells from apoptotic death. EMBO J.

[R18] Spear N, Estevez AG, Johnson GV, Bredesen DE, Thompson JA, Beckman JS (1998). Enhancement of peroxynitrite-induced apoptosis in PC12 cells by fibroblast growth factor-1 and nerve growth factor requires p21Ras activation and is suppressed by Bcl-2. Arch Biochem Biophys.

[R19] Mills EM, Takeda K, Yu ZX, Ferrans V, Katagiri Y, Jiang H, Lavigne MC, Leto TL, Guroff G (1998). Nerve growth factor treatment prevents the increase in superoxide produced by epidermal growth factor in PC12 cells. J Biol Chem.

[R20] Wei M, Fabrizio P, Madia F, Hu J, Ge H, Li LM, Longo VD (2009). Tor1/Sch9-regulated carbon source substitution is as effective as calorie restriction in life span extension. PLoS Genet.

[R21] Bluher M, Kahn BB, Kahn CR (2003). Extended longevity in mice lacking the insulin receptor in adipose tissue. Science.

[R22] Coschigano KT, Holland AN, Riders ME, List EO, Flyvbjerg A, Kopchick JJ (2003). Deletion, but not antagonism, of the mouse growth hormone receptor results in severely decreased body weights, insulin, and insulin-like growth factor I levels and increased life span. Endocrinology.

[R23] Festing MF, Blackmore DK (1971). Life span of specified-pathogen-free (MRC category 4) mice and rats. Lab Anim.

[R24] Holzenberger M, Dupont J, Ducos B, Leneuve P, Geloen A, Even PC, Cervera P, Le Bouc Y (2003). IGF-1 receptor regulates lifespan and resistance to oxidative stress in mice. Nature.

[R25] Rowlatt C, Chesterman FC, Sheriff MU (1976). Lifespan, age changes and tumour incidence in an ageing C57BL mouse colony. Lab Anim.

[R26] Storer JB (1966). Longevity and gross pathology at death in 22 inbred mouse strains. J Gerontol.

[R27] Yan L, Vatner DE, O'Connor JP, Ivessa A, Ge H, Chen W, Hirotani S, Ishikawa Y, Sadoshima J, Vatner SF (2007). Type 5 adenylyl cyclase disruption increases longevity and protects against stress. Cell.

[R28] Borras C, Sastre J, Garcia-Sala D, Lloret A, Pallardo FV, Vina J (2003). Mitochondria from females exhibit higher antioxidant gene expression and lower oxidative damage than males. Free Radic Biol Med.

[R29] Calleja M, Pena P, Ugalde C, Ferreiro C, Marco R, Garesse R (1993). Mitochondrial DNA remains intact during Drosophila aging, but the levels of mitochondrial transcripts are significantly reduced. J Biol Chem.

[R30] Crawford DR, Wang Y, Schools GP, Kochheiser J, Davies KJ (1997). Down-regulation of mammalian mitochondrial RNAs during oxidative stress. Free Radic Biol Med.

[R31] Kadenbach B, Ramzan R, Vogt S (2009). Degenerative diseases, oxidative stress and cytochrome c oxidase function. Trends Mol Med.

[R32] Timiras PS (2007). Physiological basis of aging and geriatrics.

[R33] Longo VD, Fabrizio P (2002). Regulation of longevity and stress resistance: a molecular strategy conserved from yeast to humans?. Cell Mol Life Sci.

[R34] Guarente L (2006). Sirtuins as potential targets for metabolic syndrome. Nature.

[R35] Bartke A (2008). Insulin and aging. Cell Cycle.

[R36] Bartke A (2008). New findings in gene knockout, mutant and transgenic mice. Exp Gerontol.

[R37] Bartke A (2008). Impact of reduced insulin-like growth factor-1/insulin signaling on aging in mammals: novel findings. Aging Cell.

[R38] Borras C, Stvolinsky S, Lopez-Grueso R, Fedorova T, Gambini J, Boldyrev A, Vina J (2009). Low in vivo brain glucose consumption and high oxidative stress in accelerated aging. FEBS Lett.

[R39] Ladiges W, Van Remmen H, Strong R, Ikeno Y, Treuting P, Rabinovitch P, Richardson A (2009). Lifespan extension in genetically modified mice. Aging Cell.

[R40] Selman C, Tullet JM, Wieser D, Irvine E, Lingard SJ, Choudhury AI, Claret M, Al-Qassab H, Carmignac D, Ramadani F, Woods A, Robinson IC, Schuster E, Batterham RL, Kozma SC, Thomas G, Carling D, Okkenhaug K, Thornton JM, Partridge L, Gems D, Withers DJ (2009). Ribosomal protein S6 kinase 1 signaling regulates mammalian life span. Science.

[R41] Nadon NL, Strong R, Miller RA, Nelson J, Javors M, Sharp ZD, Peralba JM, Harrison DE (2008). Design of aging intervention studies: the NIA interventions testing program. Age (Dordr).

[R42] Matheu A, Maraver A, Klatt P, Flores I, Garcia-Cao I, Borras C, Flores JM, Vina J, Blasco MA, Serrano M (2007). Delayed ageing through damage protection by the Arf/p53 pathway. Nature.

[R43] Tomas-Loba A, Flores I, Fernandez-Marcos PJ, Cayuela ML, Maraver A, Tejera A, Borras C, Matheu A, Klatt P, Flores JM, Vina J, Serrano M, Blasco MA (2008). Telomerase reverse transcriptase delays aging in cancer-resistant mice. Cell.

[R44] Gill TM, Gahbauer EA, Han L, Allore HG (2010). Trajectories of disability in the last year of life. N Engl J Med.

[R45] Studenski S, Hayes RP, Leibowitz RQ, Bode R, Lavery L, Walston J, Duncan P, Perera S (2004). Clinical Global Impression of Change in Physical Frailty: development of a measure based on clinical judgment. J Am Geriatr Soc.

[R46] Plass C, Shibata H, Kalcheva I, Mullins L, Kotelevtseva N, Mullins J, Kato R, Sasaki H, Hirotsune S, Okazaki Y, Held WA, Hayashizaki Y, Chapman VM (1996). Identification of Grf1 on mouse chromosome 9 as an imprinted gene by RLGS-M. Nat Genet.

[R47] Clapcott SJ, Peters J, Orban PC, Brambilla R, Graham CF (2003). Two ENU-induced mutations in Rasgrf1 and early mouse growth retardation. Mamm Genome.

[R48] Kawahara M, Kono T (2010). Longevity in mice without a father. Hum Reprod.

[R49] Jaenisch R (1997). DNA methylation and imprinting: why bother?. Trends Genet.

[R50] Anderson RM, Barger JL, Edwards MG, Braun KH, O'Connor CE, Prolla TA, Weindruch R (2008). Dynamic regulation of PGC-1alpha localization and turnover implicates mitochondrial adaptation in calorie restriction and the stress response. Aging Cell.

[R51] Longo VD (2009). Linking sirtuins, IGF-I signaling, and starvation. Exp Gerontol.

[R52] Gerhart-Hines Z, Rodgers JT, Bare O, Lerin C, Kim SH, Mostoslavsky R, Alt FW, Wu Z, Puigserver P (2007). Metabolic control of muscle mitochondrial function and fatty acid oxidation through SIRT1/PGC-1alpha. Embo J.

[R53] Lagouge M, Argmann C, Gerhart-Hines Z, Meziane H, Lerin C, Daussin F, Messadeq N, Milne J, Lambert P, Elliott P, Geny B, Laakso M, Puigserver P, Auwerx J (2006). Resveratrol improves mitochondrial function and protects against metabolic disease by activating SIRT1 and PGC-1alpha. Cell.

[R54] Picard F, Kurtev M, Chung N, Topark-Ngarm A, Senawong T, Machado De Oliveira R, Leid M, McBurney MW, Guarente L (2004). Sirt1 promotes fat mobilization in white adipocytes by repressing PPAR-gamma. Nature.

[R55] Rodgers JT, Lerin C, Haas W, Gygi SP, Spiegelman BM, Puigserver P (2005). Nutrient control of glucose homeostasis through a complex of PGC-1alpha and SIRT1. Nature.

[R56] Feige JN, Lagouge M, Canto C, Strehle A, Houten SM, Milne JC, Lambert PD, Mataki C, Elliott PJ, Auwerx J (2008). Specific SIRT1 activation mimics low energy levels and protects against diet-induced metabolic disorders by enhancing fat oxidation. Cell Metab.

[R57] Nakae J, Cao Y, Daitoku H, Fukamizu A, Ogawa W, Yano Y, Hayashi Y (2006). The LXXLL motif of murine forkhead transcription factor FoxO1 mediates Sirt1-dependent transcriptional activity. J Clin Invest.

[R58] Luptak I, Yan J, Cui L, Jain M, Liao R, Tian R (2007). Long-term effects of increased glucose entry on mouse hearts during normal aging and ischemic stress. Circulation.

[R59] Wessells RJ, Fitzgerald E, Cypser JR, Tatar M, Bodmer R (2004). Insulin regulation of heart function in aging fruit flies. Nat Genet.

[R60] Longo VD (2003). The Ras and Sch9 pathways regulate stress resistance and longevity. Exp Gerontol.

[R61] Longo VD (2004). Ras: the other pro-aging pathway. Sci Aging Knowledge Environ 2004.

[R62] Sebastiani P, Solovieff N, Puca A, Hartley SW, Melista E, Andersen S, Dworkis DA, Wilk JB, Myers RH, Steinberg MH, Montano M, Baldwin CT, Perls TT (2010). Genetic Signatures of Exceptional Longevity in Humans. Science.

[R63] Ingram DK, Reynolds MA (1986). Assessing the predictive validity of psychomotor tests as measures of biological age in mice. Exp Aging Res.

[R64] Asensi M, Sastre J, Pallardo FV, Estrela JM, Vina J (1994). Determination of oxidized glutathione in blood: high-performance liquid chromatography. Methods Enzymol.

[R65] Wong SH, Knight JA, Hopfer SM, Zaharia O, Leach CN, Sunderman FW (1987). Lipoperoxides in plasma as measured by liquid-chromatographic separation of malondialdehyde-thiobarbituric acid adduct. Clin Chem.

[R66] Fueger BJ, Czernin J, Hildebrandt I, Tran C, Halpern BS, Stout D, Phelps ME, Weber WA (2006). Impact of animal handling on the results of 18F-FDG PET studies in mice. J Nucl Med.

[R67] Hamacher K, Coenen HH, Stocklin G (1986). Efficient stereospecific synthesis of no-carrier-added 2-[18F]-fluoro-2-deoxy-D-glucose using aminopolyether supported nucleophilic substitution. J Nucl Med.

[R68] Trygg J, Holmes E, Lundstedt T (2007). Chemometrics in metabonomics. J Proteome Res.

