# Supplementary material for: RasGrf1 deficiency delays aging in mice
Source: Aging (Albany NY). 2011 Mar 14;3(3):262–76. doi: 10.18632/aging.100279 (PMC3091520; doi:10.18632/aging.100279)
Supplement: Supplementary file 1 [file aging-03-262-s001.pdf]

SUPPLEMENTARY MATERIAL

Table for data of Figure 1

Gender	Animal ID	*RasGrf1*	D.O.B.	D.O.D.	Weeks alive		
♂	705-2	KO	5/6/2007	8/1/2007	12.428571		
♂	302-2	KO	12/12/2005	10/23/2006	45		
♂	37-1	KO	1/28/2005	7/31/2006	78.428571		
♂	505-3(w)	KO	8/21/2006	2/23/2008	78.714286		
♂	92-1	KO	5/17/2005	12/18/2006	82.857143		
♂	173-1	KO	9/6/2005	6/11/2007	91.857143		
♂	274-1	KO	11/22/2005	9/1/2007	92.571429		
♂	5-5	KO	1/10/2005	11/5/2006	94.857143		
♂	427-2	KO	4/16/2006	3/6/2008	98.571429		
♂	504-2(w)	KO	8/21/2006	7/22/2008	100.14286		
♂	228-1	KO	10/18/2005	9/28/2007	101.42857		
♂	426-1	KO	4/16/2006	3/31/2008	102.14286		
♂	62-2	KO	2/21/2005	2/23/2007	104.57143		
♂	481-2(w)	KO	7/9/2006	8/5/2008	108.28571		
♂	611-2	KO	1/22/2007	2/19/2009	108.42857		
♂	699-1	KO	4/30/2007	6/20/2009	111.71429		
♂	389-2	KO	3/6/2006	4/27/2008	111.85714		
♂	610-1	KO	1/22/2007	3/19/2009	112.42857		
♂	625-1	KO	2/20/2007	5/15/2009	116.42857		
♂	303-3	KO	12/12/2005	3/20/2008	118.42857		
♂	701-3	KO	4/30/2007	8/15/2009	119.71429		
♂	480-1-(w)	KO	7/9/2006	11/7/2008	121.71429		
♂	190-2	KO	9/28/2005	2/10/2008	123.57143		
♂	182-1	KO	9/23/2005	2/15/2008	125		
♂	191-3	KO	9/28/2005	2/23/2008	125.42857		
♂	J9-1	KO	1/20/2005	7/6/2007	128.14286		
♂	64-1	KO	2/22/2005	9/7/2007	132.42857		
♂	231-1	KO	9/28/2005	4/17/2008	133.14286		
♂	48-1	KO	3/3/2005	9/27/2007	134		
♂	503-1(w)	KO	8/21/2006	5/5/2009	141.14286		
♂	230-1(w)	KO	9/28/2005	6/17/2008	141.85714	**TUMOR**	Colon tumor
♂	479-1(b)	KO	7/9/2006	4/19/2009	145		
♂	40-1	KO	3/28/2005	1/8/2008	145.14286		
♂	251-1	KO	11/16/2005	8/30/2008	145.42857	**TUMOR**	Chest wall tumor
♂	J3-3	KO	1/10/2005	10/26/2007	145.57143	**TUMOR**	Fast growing tumor in the front right leg
♂	65-2	KO	2/22/2005	12/16/2007	146.71429		
♂	181-1	KO	9/19/2005	7/31/2008	149.42857		

♂	482-3(w)	KO	7/9/2006	5/23/2009	149.85714
♂	167-2	KO	8/28/2005	7/13/2008	150
♂	63-3	KO	2/21/2005	1/8/2008	150.14286
♂	140-3	KO	7/12/2005	6/11/2008	152.14286
♂	275-2	KO	11/22/2005	12/3/2008	158.14286
♂	J33-2(alb)	KO	1/27/2005	2/9/2008	158.28571
♂	43-4	KO	3/28/2005	5/13/2008	163.14286
♂	300-1 (w)	KO	12/12/2005	4/13/2009	174

SUPPLEMENTARY FIGURES

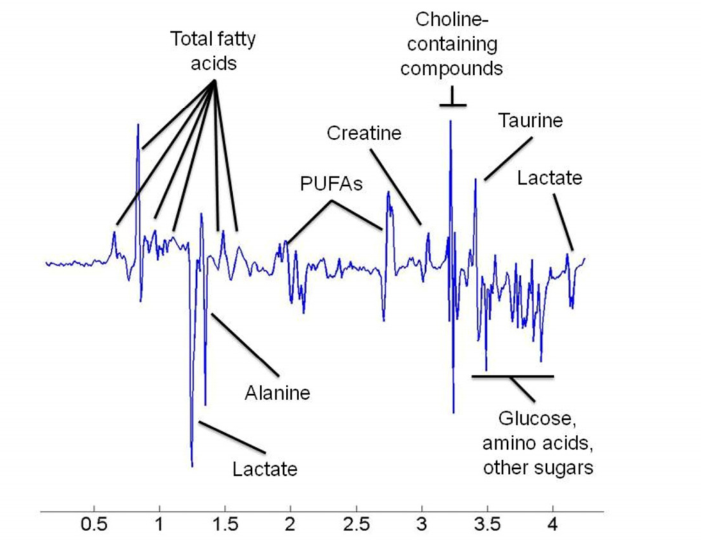

Figure S1. Loadings Plot for The First Latent Variable of The PLSDA Model for Discrimination Between *RasGrf1$^{-/-}$* and WT male mice. The plot illustrates the contributions of most relevant metabolites (for assignments see Nicholson et al., 1995). Positive values over the baseline in this loading plot mean an increase in the level of the metabolite parallel to an increase in the value of the latent variable. For instance, WT mice in calorie restriction (gray triangles in figure 1c) have higher values of LV1 than WT with normal diet (gray circles in figure 1c). This means that WT mice in calorie restriction have higher levels of global fatty acids (positive value in this loadings plot) and lower levels of lactate (negative value in this loadings plot) than WT with normal diet.

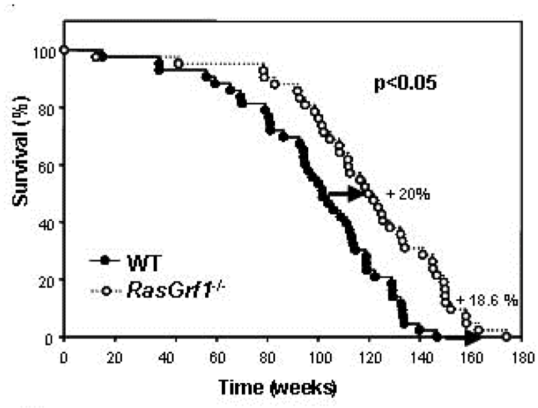

Figure S2. Survival curve without tumours. Survival curve of WT n=43 and *RasGrf1$^{-/-}$* n=42 male mice, same group of animals as in Figure 1, but excluding those animals that presented tumours at the time of death ($p<0.05$).

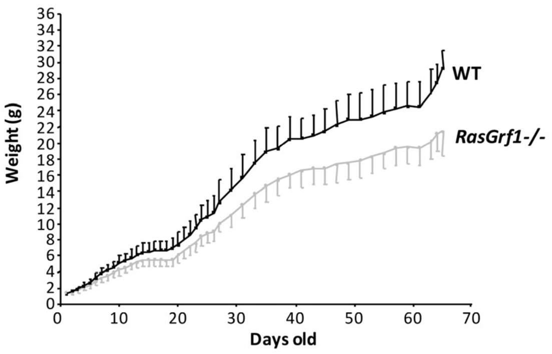

Figure S3. Body weight curves of WT and *RasGrf1$^{-/-}$* male mice. Data are reported as mean weight ± SEM (n=24 for WT, n=19 for *RasGrf1$^{-/-}$*).

SUPPLEMENTARY TABLE

Table S1. Procedure 055: Monitor of Aging Mice "Regulation of Longevity in RasGrf1 ko Mice"

Initial date of observation: Weight:

ID number of Cage/Animal:

Current weight								
				Date				
Parameters			Punctuation					
Body Weight Loss		None	0					
		5-10%	9					
		10-15%	12					
		>15%	18					
General Appearance		Normal	0					
		Loss of activity, lack of grooming	2					
		Poor hair condition	3					
		Dehydrated, inactivity, hunched posture (lordosis)	6					
		Moribund or cachexia	18					
		Derange vital functions*	18					
		Ascitis and/or abdominal distention	10					
Tumors	Size	<7 mm	0					
		7-17mm	9					
		>18mm/abdominal distention (>10% weight)	12					
		Skin ulcer	18					
Wound	Surfa-ce (S^2)	0-5% S^2 body	7					
		5-10% S^2 body	12					
		>10% S^2 body	18					
	Tissu e	Subcutaneous	3					
		Muscular	5					
		Mucosa	12					
Prolapses	Locali-zation	Penil	6					
		Rectal, vaginal- uterine	9					
		Ocular	18					
	Muco-sal	normal	0					
		congestive	9					
		necrosia	18					
FINAL PUNCTUATION								
SIGNATURE								

***Vital functions:** afflicting one of the 5 senses and physiological functions (food ingestion, urinate, defecate and motility)

0-7: daily monitoring

8-13: seek advice with veterinarians and plan for treatment (first aid, analgesics, etc.)

14-17: seek advice with veterinarians and plan for euthanasia

≥ 18: immediate euthanasia
